# Supplementary material for: Little evidence for an effect of smoking on multiple sclerosis risk: A Mendelian Randomization study
Source: PLoS Biol. 2020 Nov 30;18(11):e3000973. doi: 10.1371/journal.pbio.3000973 (PMC7728259; doi:10.1371/journal.pbio.3000973)
Supplement: S1 Data — (DOCX) [file pbio.3000973.s001.docx]

**Little evidence for an effect of smoking on multiple sclerosis risk: a Mendelian Randomization study**

Ruth E Mitchell, Kirsty Bates, Robyn E Wootton, Adil Harroud, J. Brent Richards, George Davey Smith, Marcus R Munafò

SUPPLEMENTARY MATERIAL

SI Fig A. Scatter plot of inverse variance weighted Mendelian Randomisation and sensitivity analyses of smoking initiation on susceptibility of multiple sclerosis

Scatter plot showing the relationship of SNP-smoking initiation association (x-axis, SD units) against the SNP-multiple sclerosis association (y-axis, log OR). Each of the SNPs associated with smoking initiation is represented by a black dot with the error bar depicting the standard error of its association with smoking initiation (horizontal) and multiple sclerosis incidence (vertical). The slopes of the lines correspond to the causal estimates as calculated using five different MR methods. The genetic variants used to proxy smoking initiation are the conditionally independent genome-wide significant SNPs taken from the GWAS & Sequencing Consortium of Alcohol and Nicotine use (GSCAN) consortium detailed in Table A in S1 Data. The estimates of their association with MS are taken from the 2019 MS Chip International Multiple Sclerosis Genetics Consortium (IMSGC) meta-analysis.

SI Fig B. Funnel plot for the effect of smoking initiation on susceptibility of multiple sclerosis

Funnel plot showing the relationship between the causal effect of smoking initiation on susceptibility of multiple sclerosis estimated using each individual SNP as a separate instrument against the inverse of the standard error of the causal estimate. Vertical lines show the causal estimates using all SNPs combined into a single instrument for the inverse variance weighted and MR Egger methods. The genetic variants used to proxy smoking initiation are the conditionally independent genome-wide significant SNPs taken from the GWAS & Sequencing Consortium of Alcohol and Nicotine use (GSCAN) consortium detailed in Table A in S1 Data. The estimates of their association with MS are taken from the 2019 MS Chip International Multiple Sclerosis Genetics Consortium (IMSGC) meta-analysis.

SI Fig C. Leave-one-out sensitivity analysis of the causal effect of smoking initiation on susceptibility of multiple sclerosis

A forest plot where each black point represents the inverse variance weighted (IWV) MR method applied to estimate the causal effect of smoking initiation on susceptibility of multiple sclerosis excluding that particular variant from the analysis. The red point depicts the IVW estimate using all SNPs. Horizontal lines denote 95% confidence intervals. The genetic variants used to proxy smoking initiation are the conditionally independent genome-wide significant SNPs taken from the GWAS & Sequencing Consortium of Alcohol and Nicotine use (GSCAN) consortium detailed in Table A in S1 Data. The estimates of their association with MS are taken from the 2019 MS Chip International Multiple Sclerosis Genetics Consortium (IMSGC) meta-analysis.

SI Fig D. Single SNP sensitivity analysis of the causal effect of smoking initiation on susceptibility of multiple sclerosis

A forest plot where each black point represents the inverse variance weighted (IWV) MR method applied to estimate the causal effect of smoking initiation on susceptibility of multiple sclerosis using only that particular variant. The red point depicts the IVW estimate using all SNPs. Horizontal lines denote 95% confidence intervals. The genetic variants used to proxy smoking initiation are the conditionally independent genome-wide significant SNPs taken from the GWAS & Sequencing Consortium of Alcohol and Nicotine use (GSCAN) consortium detailed in Table A in S1 Data. The estimates of their association with MS are taken from the 2019 MS Chip International Multiple Sclerosis Genetics Consortium (IMSGC) meta-analysis.

SI Fig E. Scatter plot of inverse variance weighted Mendelian Randomisation and sensitivity analyses of lifetime smoking on susceptibility of multiple sclerosis

Scatter plot showing the relationship of SNP-lifetime smoking association (x-axis, SD units) against the SNP-multiple sclerosis association (y-axis, log OR). Each of the SNPs associated with lifetime smoking is represented by a black dot with the error bar depicting the standard error of its association with lifetime smoking (horizontal) and multiple sclerosis incidence (vertical). The slopes of the lines correspond to the causal estimates as calculated using five different MR methods. The genetic variants used to proxy lifetime smoking are the independent genome-wide significant SNPs taken from the GWAS of lifetime smoking performed by Wootton et al. 2019 detailed in Table C in S1 Data. The estimates of their association with MS are taken from the 2019 MS Chip International Multiple Sclerosis Genetics Consortium (IMSGC) meta-analysis.

SI Fig F. Funnel plot for the effect of lifetime smoking on susceptibility of multiple sclerosis

Funnel plot showing the relationship between the causal effect of lifetime smoking on susceptibility of multiple sclerosis estimated using each individual SNP as a separate instrument against the inverse of the standard error of the causal estimate. Vertical lines show the causal estimates using all SNPs combined into a single instrument for the inverse variance weighted and MR Egger methods. The genetic variants used to proxy lifetime smoking are the independent genome-wide significant SNPs taken from the GWAS of lifetime smoking performed by Wootton et al. 2019 detailed in Table C in S1 Data. The estimates of their association with MS are taken from the 2019 MS Chip International Multiple Sclerosis Genetics Consortium (IMSGC) meta-analysis.

SI Fig G. Leave-one-out sensitivity analysis of the causal effect of lifetime smoking on susceptibility of multiple sclerosis

A forest plot where each black point represents the inverse variance weighted (IWV) MR method applied to estimate the causal effect of lifetime smoking on susceptibility of multiple sclerosis excluding that particular variant from the analysis. The red point depicts the IVW estimate using all SNPs. Horizontal lines denote 95% confidence intervals. The genetic variants used to proxy lifetime smoking are the independent genome-wide significant SNPs taken from the GWAS of lifetime smoking performed by Wootton et al. 2019 detailed in Table C in S1 Data. The estimates of their association with MS are taken from the 2019 MS Chip International Multiple Sclerosis Genetics Consortium (IMSGC) meta-analysis.

SI Fig H. Single SNP sensitivity analysis of the causal effect of lifetime smoking on susceptibility of multiple sclerosis

A forest plot where each black point represents the inverse variance weighted (IWV) MR method applied to estimate the causal effect of lifetime smoking on susceptibility of multiple sclerosis using only that particular variant. The red point depicts the IVW estimate using all SNPs. Horizontal lines denote 95% confidence intervals. The genetic variants used to proxy lifetime smoking are the independent genome-wide significant SNPs taken from the GWAS of lifetime smoking performed by Wootton et al. 2019 detailed in Table C in S1 Data. The estimates of their association with MS are taken from the 2019 MS Chip International Multiple Sclerosis Genetics Consortium (IMSGC) meta-analysis.

SUPPLEMENTARY TABLES

SI Table A: List of independent SNPs associated with smoking initiation at the genome-wide level of significance (p<5x10-8) used as instruments in the Mendelian randomization analysis.

Genetic variants and effect sizes for smoking initiation from Liu et al meta-analysis^1^

SNP: Single Nucleotide Polymorphism; CHR: Chromosome; BP: Base pair position of the variant according to human reference sequence (GRCh37), Hg19; EA: Effect Allele; OA: Other Allele; EAF: Effect Allele Frequency; beta per effect allele; Standard error of the beta; p value: Strength of evidence against the null hypothesis of no association between variant and outcome.

| **SNP** | **Original SNP if proxy used in analysis** | **CHR** | **Base pair**  **Position** | **EA** | **OA** | **EAF** | **beta** | **Stan-**  **dard**  **Error** | **p value** |
| --- | --- | --- | --- | --- | --- | --- | --- | --- | --- |
| rs12130857 |  | 1 | 7791461 | A | G | 0.32 | -0.018 | 0.003 | 3.65E-11 |
| rs301807 |  | 1 | 8484823 | G | A | 0.57 | 0.018 | 0.003 | 2.50E-12 |
| rs3820277 |  | 1 | 18436657 | T | G | 0.53 | -0.019 | 0.003 | 1.57E-13 |
| rs1889571 |  | 1 | 32195819 | G | T | 0.13 | 0.022 | 0.004 | 4.19E-09 |
| rs10914684 |  | 1 | 33795572 | A | G | 0.32 | -0.016 | 0.003 | 6.32E-09 |
| rs2637869 |  | 1 | 38757237 | A | G | 0.30 | 0.018 | 0.003 | 6.54E-11 |
| rs12755632 |  | 1 | 41776623 | G | A | 0.32 | -0.015 | 0.003 | 1.93E-08 |
| rs951740 |  | 1 | 44011737 | A | G | 0.63 | 0.030 | 0.003 | 3.82E-29 |
| rs925524 |  | 1 | 46496709 | G | A | 0.71 | 0.016 | 0.003 | 2.94E-08 |
| rs12022778 |  | 1 | 50603995 | C | A | 0.20 | 0.027 | 0.003 | 3.18E-17 |
| rs11587399 |  | 1 | 50861071 | T | A | 0.22 | -0.018 | 0.003 | 7.25E-09 |
| rs4912332 |  | 1 | 58815243 | T | C | 0.49 | 0.014 | 0.003 | 2.94E-08 |
| rs1937443 |  | 1 | 66469643 | G | C | 0.56 | 0.020 | 0.003 | 1.79E-15 |
| rs1022528 |  | 1 | 71490122 | A | G | 0.34 | 0.017 | 0.003 | 8.48E-11 |
| rs12740789 |  | 1 | 72752073 | A | G | 0.18 | -0.028 | 0.003 | 1.18E-17 |
| rs992956 | rs80054503 | 1 | 72901141 | G | T | 0.12 | -0.024 | 0.004 | 3.10E-09 |
| rs10789369 |  | 1 | 73824909 | G | A | 0.62 | -0.023 | 0.003 | 3.39E-19 |
| rs1514176 |  | 1 | 74991596 | A | G | 0.58 | -0.019 | 0.003 | 7.67E-14 |
| rs10873871 |  | 1 | 76689019 | G | A | 0.21 | 0.017 | 0.003 | 2.82E-08 |
| rs11162019 |  | 1 | 87913176 | T | C | 0.36 | -0.015 | 0.003 | 5.06E-09 |
| rs1008078 |  | 1 | 91189731 | T | C | 0.40 | 0.023 | 0.003 | 1.63E-18 |
| rs1935571 |  | 1 | 96414335 | G | T | 0.48 | -0.016 | 0.003 | 6.99E-10 |
| rs12027999 |  | 1 | 154206358 | C | T | 0.12 | -0.024 | 0.004 | 5.33E-10 |
| rs45444697 |  | 1 | 155034632 | G | C | 0.21 | 0.020 | 0.003 | 2.72E-10 |
| rs2901785 |  | 1 | 174104743 | A | G | 0.45 | -0.017 | 0.003 | 1.47E-11 |
| rs147052174 |  | 1 | 179783167 | T | G | 0.02 | 0.062 | 0.010 | 2.30E-10 |
| rs35656245 |  | 1 | 190957480 | A | G | 0.28 | 0.016 | 0.003 | 2.23E-08 |
| rs12739243 |  | 1 | 210302043 | C | T | 0.22 | -0.021 | 0.003 | 4.45E-12 |
| rs12563365 |  | 1 | 236872829 | A | G | 0.56 | 0.017 | 0.003 | 1.05E-10 |
| rs876793 |  | 1 | 237852083 | C | T | 0.35 | -0.018 | 0.003 | 5.69E-11 |
| rs114976176 |  | 2 | 264621 | C | A | 0.35 | -0.016 | 0.003 | 6.04E-09 |
| rs6731872 |  | 2 | 624205 | G | T | 0.83 | 0.032 | 0.003 | 5.35E-21 |
| rs1022376 |  | 2 | 22067213 | C | T | 0.52 | -0.015 | 0.003 | 1.66E-08 |
| rs61533748 |  | 2 | 22582968 | C | T | 0.38 | 0.017 | 0.003 | 2.82E-11 |
| rs72790288 |  | 2 | 29513404 | A | G | 0.03 | -0.046 | 0.008 | 3.28E-09 |
| rs2710634 |  | 2 | 32808804 | C | T | 0.52 | -0.018 | 0.003 | 3.36E-12 |
| rs62137126 |  | 2 | 44250149 | G | A | 0.12 | -0.024 | 0.004 | 1.31E-09 |
| rs1004787 |  | 2 | 45159091 | A | G | 0.55 | 0.028 | 0.003 | 1.11E-28 |
| rs7598402 |  | 2 | 50735943 | G | C | 0.49 | -0.015 | 0.003 | 7.38E-09 |
| rs10490159 |  | 2 | 51341259 | T | C | 0.39 | 0.017 | 0.003 | 3.86E-11 |
| rs1518393 |  | 2 | 58171220 | C | A | 0.62 | 0.017 | 0.003 | 1.30E-10 |
| rs17616642 |  | 2 | 59022210 | G | A | 0.25 | -0.017 | 0.003 | 2.10E-08 |
| rs6730325 |  | 2 | 59315828 | A | G | 0.61 | -0.015 | 0.003 | 2.10E-08 |
| rs2539706 |  | 2 | 59819545 | A | G | 0.53 | 0.016 | 0.003 | 1.95E-10 |
| rs7585579 |  | 2 | 60024857 | G | C | 0.50 | 0.020 | 0.003 | 5.48E-15 |
| rs1863161 |  | 2 | 60139524 | A | G | 0.56 | 0.015 | 0.003 | 2.34E-09 |
| rs359247 |  | 2 | 60477052 | T | A | 0.64 | 0.022 | 0.003 | 9.89E-17 |
| rs62180324 |  | 2 | 63416606 | A | G | 0.21 | -0.020 | 0.003 | 3.91E-10 |
| rs6750107 |  | 2 | 80748807 | A | G | 0.39 | 0.015 | 0.003 | 2.60E-08 |
| rs12714017 |  | 2 | 80999398 | C | T | 0.51 | 0.015 | 0.003 | 3.65E-09 |
| rs56208390 |  | 2 | 83247997 | G | A | 0.12 | 0.022 | 0.004 | 2.68E-08 |
| rs11692435 |  | 2 | 98275354 | A | G | 0.08 | 0.025 | 0.005 | 4.47E-08 |
| rs13392222 |  | 2 | 100672408 | C | A | 0.14 | -0.023 | 0.004 | 1.93E-10 |
| rs1901477 |  | 2 | 104126983 | G | A | 0.51 | 0.030 | 0.003 | 2.07E-31 |
| rs11889814 |  | 2 | 104432494 | C | A | 0.13 | -0.021 | 0.004 | 3.44E-08 |
| rs3811038 |  | 2 | 113240183 | C | T | 0.28 | 0.019 | 0.003 | 1.58E-11 |
| rs75210106 |  | 2 | 113246436 | T | C | 0.18 | -0.019 | 0.003 | 2.33E-08 |
| rs34399632 |  | 2 | 137571174 | G | A | 0.23 | 0.019 | 0.003 | 1.46E-10 |
| rs74697736 |  | 2 | 145412271 | A | G | 0.29 | 0.022 | 0.003 | 2.43E-15 |
| rs6756212 |  | 2 | 146140132 | T | C | 0.54 | -0.034 | 0.003 | 3.49E-40 |
| rs16826827 |  | 2 | 147825689 | C | T | 0.12 | -0.022 | 0.004 | 9.17E-09 |
| rs1445649 |  | 2 | 155682556 | C | T | 0.54 | 0.021 | 0.003 | 8.48E-16 |
| rs1722666 |  | 2 | 161816880 | T | C | 0.73 | 0.016 | 0.003 | 2.17E-08 |
| rs11678980 |  | 2 | 162101261 | A | G | 0.45 | 0.018 | 0.003 | 5.19E-12 |
| rs12474587 |  | 2 | 162802993 | T | G | 0.43 | 0.024 | 0.003 | 4.83E-21 |
| rs357304 |  | 2 | 164862639 | C | T | 0.73 | 0.017 | 0.003 | 5.40E-09 |
| rs13007361 |  | 2 | 166250244 | A | G | 0.21 | 0.018 | 0.003 | 2.29E-08 |
| rs7600835 |  | 2 | 172521827 | A | G | 0.34 | -0.015 | 0.003 | 1.80E-08 |
| rs6750529 |  | 2 | 182027603 | T | C | 0.74 | 0.020 | 0.003 | 9.26E-12 |
| rs17229285 |  | 2 | 199523122 | T | C | 0.51 | -0.015 | 0.003 | 1.27E-09 |
| rs3115418 |  | 2 | 200936399 | C | T | 0.45 | -0.014 | 0.003 | 2.79E-08 |
| rs62193862 |  | 2 | 202843875 | A | G | 0.10 | 0.024 | 0.004 | 1.99E-08 |
| rs4674916 |  | 2 | 225365635 | A | C | 0.33 | -0.018 | 0.003 | 3.06E-11 |
| rs4674993 |  | 2 | 226332033 | G | A | 0.20 | -0.024 | 0.003 | 4.85E-14 |
| rs11713899 |  | 3 | 2365026 | C | A | 0.17 | 0.019 | 0.003 | 3.15E-08 |
| rs748832 |  | 3 | 16851202 | G | A | 0.37 | 0.017 | 0.003 | 6.60E-11 |
| rs10446419 |  | 3 | 25725501 | G | A | 0.21 | -0.020 | 0.003 | 5.05E-10 |
| rs13319205 |  | 3 | 47800216 | A | T | 0.29 | 0.017 | 0.003 | 3.77E-09 |
| rs3172494 |  | 3 | 48731487 | T | G | 0.12 | -0.029 | 0.004 | 3.40E-13 |
| rs2526390 |  | 3 | 50192760 | T | C | 0.33 | 0.020 | 0.003 | 3.62E-14 |
| rs2276825 |  | 3 | 52886605 | C | T | 0.25 | 0.019 | 0.003 | 1.89E-10 |
| rs2306866 |  | 3 | 53766212 | T | A | 0.61 | -0.017 | 0.003 | 1.89E-10 |
| rs73831818 |  | 3 | 55988394 | G | A | 0.06 | 0.032 | 0.005 | 5.46E-09 |
| rs1910236 |  | 3 | 59434420 | A | G | 0.47 | 0.015 | 0.003 | 9.91E-09 |
| rs7640107 |  | 3 | 59966156 | T | C | 0.43 | -0.014 | 0.003 | 3.46E-08 |
| rs2734390 |  | 3 | 60459291 | G | A | 0.37 | 0.015 | 0.003 | 2.09E-08 |
| rs221988 |  | 3 | 64234307 | C | A | 0.38 | -0.015 | 0.003 | 1.43E-08 |
| rs2196356 |  | 3 | 70890288 | C | G | 0.29 | -0.019 | 0.003 | 2.45E-11 |
| rs11128203 |  | 3 | 71064431 | A | T | 0.53 | 0.020 | 0.003 | 1.29E-15 |
| rs62246017 |  | 3 | 71483084 | A | G | 0.32 | -0.016 | 0.003 | 3.03E-09 |
| rs4543050 |  | 3 | 74954560 | T | A | 0.82 | 0.022 | 0.003 | 1.45E-11 |
| rs6782116 |  | 3 | 77176032 | T | C | 0.42 | -0.015 | 0.003 | 1.46E-08 |
| rs13066050 |  | 3 | 81325861 | T | C | 0.21 | 0.019 | 0.003 | 1.93E-09 |
| rs12633090 |  | 3 | 83241365 | C | G | 0.18 | -0.023 | 0.003 | 3.16E-12 |
| rs1549979 |  | 3 | 85460131 | T | C | 0.62 | -0.025 | 0.003 | 8.80E-21 |
| rs11922956 | rs74664784 | 3 | 85497807 | G | A | 0.38 | -0.020 | 0.003 | 9.34E-13 |
| rs57153235 |  | 3 | 85902536 | G | T | 0.32 | -0.019 | 0.003 | 1.56E-12 |
| rs6437769 |  | 3 | 107997514 | T | C | 0.58 | 0.014 | 0.003 | 3.74E-08 |
| rs9288999 |  | 3 | 114147927 | A | G | 0.74 | 0.017 | 0.003 | 1.50E-09 |
| rs6438436 |  | 3 | 117822149 | T | C | 0.82 | 0.025 | 0.003 | 5.33E-14 |
| rs12053870 |  | 3 | 118302515 | G | T | 0.54 | 0.016 | 0.003 | 1.02E-09 |
| rs9826984 |  | 3 | 131945722 | A | G | 0.54 | -0.014 | 0.003 | 3.87E-08 |
| rs2279829 |  | 3 | 147106319 | T | C | 0.22 | -0.017 | 0.003 | 2.05E-08 |
| rs2319545 |  | 3 | 147719648 | A | C | 0.15 | 0.023 | 0.004 | 8.30E-11 |
| rs10935779 |  | 3 | 149543102 | T | C | 0.42 | -0.014 | 0.003 | 2.95E-08 |
| rs963354 |  | 3 | 157393770 | A | C | 0.69 | 0.015 | 0.003 | 4.21E-08 |
| rs1714521 |  | 3 | 158284861 | C | A | 0.41 | -0.016 | 0.003 | 3.07E-10 |
| rs1449012 |  | 3 | 159048333 | T | C | 0.46 | -0.015 | 0.003 | 1.77E-09 |
| rs9850597 |  | 3 | 161761866 | A | G | 0.82 | -0.019 | 0.003 | 1.65E-08 |
| rs1187820 |  | 3 | 173072584 | T | C | 0.44 | -0.014 | 0.003 | 2.69E-08 |
| rs16828799 |  | 3 | 173353739 | T | G | 0.16 | 0.020 | 0.004 | 1.83E-08 |
| rs9841807 |  | 3 | 175718927 | T | C | 0.27 | 0.016 | 0.003 | 1.35E-08 |
| rs7631379 |  | 3 | 181409057 | C | T | 0.21 | 0.021 | 0.003 | 3.94E-11 |
| rs4140932 |  | 4 | 15458598 | A | T | 0.43 | -0.014 | 0.003 | 4.89E-08 |
| rs12642744 |  | 4 | 28027176 | T | G | 0.74 | -0.017 | 0.003 | 2.82E-08 |
| rs59537158 |  | 4 | 28246049 | T | C | 0.21 | 0.022 | 0.003 | 4.62E-13 |
| rs1389171 |  | 4 | 28822284 | A | T | 0.24 | -0.017 | 0.003 | 4.45E-09 |
| rs55944129 |  | 4 | 29082156 | C | T | 0.27 | -0.018 | 0.003 | 1.06E-09 |
| rs58400863 |  | 4 | 31184484 | A | G | 0.35 | -0.020 | 0.003 | 4.89E-14 |
| rs7657022 |  | 4 | 35501032 | G | A | 0.49 | 0.018 | 0.003 | 7.34E-13 |
| rs1399119 | rs55900829 | 4 | 35514938 | A | G | 0.33 | 0.019 | 0.003 | 5.63E-12 |
| rs112725451 |  | 4 | 68017710 | T | C | 0.17 | 0.026 | 0.003 | 1.65E-14 |
| rs1160685 |  | 4 | 94052854 | G | C | 0.45 | 0.015 | 0.003 | 2.31E-09 |
| rs1435479 |  | 4 | 94550450 | T | G | 0.29 | 0.016 | 0.003 | 5.68E-09 |
| rs3934797 |  | 4 | 112467612 | A | G | 0.18 | -0.021 | 0.003 | 1.12E-10 |
| rs71602617 |  | 4 | 136406155 | T | C | 0.22 | -0.018 | 0.003 | 2.10E-08 |
| rs7696257 |  | 4 | 137474783 | A | G | 0.37 | 0.015 | 0.003 | 6.78E-09 |
| rs13109980 |  | 4 | 140886963 | A | G | 0.33 | -0.022 | 0.003 | 3.37E-16 |
| rs1116690 |  | 4 | 143510148 | G | A | 0.74 | 0.016 | 0.003 | 2.16E-08 |
| rs13110073 |  | 4 | 147797913 | C | T | 0.40 | -0.025 | 0.003 | 3.24E-21 |
| rs28717373 |  | 4 | 147985231 | T | C | 0.36 | -0.016 | 0.003 | 6.16E-10 |
| rs62340589 |  | 4 | 176875795 | C | G | 0.20 | 0.017 | 0.003 | 4.31E-08 |
| rs12517438 |  | 5 | 30842054 | G | T | 0.54 | 0.015 | 0.003 | 1.89E-09 |
| rs35375873 |  | 5 | 43190647 | C | G | 0.11 | -0.027 | 0.004 | 3.29E-11 |
| rs986714 |  | 5 | 50821338 | T | A | 0.45 | -0.016 | 0.003 | 4.13E-10 |
| rs71592686 |  | 5 | 60121271 | C | T | 0.27 | 0.021 | 0.003 | 3.85E-13 |
| rs2028269 |  | 5 | 79308315 | A | G | 0.40 | 0.016 | 0.003 | 5.19E-10 |
| rs6874731 |  | 5 | 80263865 | G | T | 0.48 | 0.015 | 0.003 | 1.83E-09 |
| rs6452785 |  | 5 | 87685500 | T | C | 0.47 | -0.027 | 0.003 | 4.69E-26 |
| rs10805858 |  | 5 | 88873832 | T | A | 0.34 | 0.018 | 0.003 | 1.88E-11 |
| rs42417 |  | 5 | 94198290 | T | C | 0.69 | 0.017 | 0.003 | 8.27E-10 |
| rs72780746 |  | 5 | 103929588 | C | T | 0.17 | -0.026 | 0.003 | 2.05E-14 |
| rs10060196 |  | 5 | 106455988 | A | C | 0.58 | 0.018 | 0.003 | 1.29E-12 |
| rs72789626 |  | 5 | 106825618 | A | T | 0.14 | -0.026 | 0.004 | 5.13E-12 |
| rs17165769 |  | 5 | 107365642 | G | A | 0.39 | 0.016 | 0.003 | 9.56E-10 |
| rs329124 |  | 5 | 133865452 | G | A | 0.43 | -0.016 | 0.003 | 1.96E-10 |
| rs1385108 |  | 5 | 154839646 | T | C | 0.24 | 0.019 | 0.003 | 3.84E-10 |
| rs1173461 |  | 5 | 157707571 | T | C | 0.33 | 0.017 | 0.003 | 9.51E-10 |
| rs11956866 |  | 5 | 161018271 | G | T | 0.57 | -0.015 | 0.003 | 7.82E-09 |
| rs3909281 |  | 5 | 165096435 | G | T | 0.54 | 0.021 | 0.003 | 1.62E-16 |
| rs3843905 |  | 5 | 165427280 | T | C | 0.40 | -0.015 | 0.003 | 5.41E-09 |
| rs79476395 |  | 5 | 166063680 | G | A | 0.07 | 0.033 | 0.005 | 1.04E-11 |
| rs6890961 |  | 5 | 166778503 | T | C | 0.62 | -0.019 | 0.003 | 2.13E-13 |
| rs4044321 |  | 5 | 166989513 | G | A | 0.64 | -0.023 | 0.003 | 1.75E-17 |
| rs2173019 |  | 5 | 167614971 | A | T | 0.18 | 0.028 | 0.003 | 2.98E-17 |
| rs10042827 |  | 5 | 170299916 | C | T | 0.68 | 0.017 | 0.003 | 9.41E-10 |
| rs359431 |  | 5 | 173288534 | T | C | 0.56 | -0.014 | 0.003 | 3.16E-08 |
| rs1059490 |  | 6 | 26171250 | C | T | 0.37 | -0.019 | 0.003 | 2.16E-12 |
| rs6932350 |  | 6 | 26571629 | A | T | 0.45 | 0.015 | 0.003 | 5.13E-09 |
| rs1150668 |  | 6 | 28129789 | G | T | 0.42 | -0.019 | 0.003 | 8.54E-13 |
| rs1632941 |  | 6 | 29796685 | C | T | 0.46 | -0.016 | 0.003 | 6.67E-10 |
| rs3218116 |  | 6 | 41901763 | T | C | 0.26 | -0.020 | 0.003 | 1.05E-11 |
| rs160631 |  | 6 | 52895230 | G | T | 0.73 | -0.017 | 0.003 | 1.87E-09 |
| rs7743165 |  | 6 | 67521222 | G | T | 0.50 | 0.019 | 0.003 | 4.15E-14 |
| rs12212564 | rs79180767 | 6 | 67540417 | T | C | 0.25 | 0.020 | 0.003 | 7.00E-12 |
| rs10945141 |  | 6 | 69470709 | A | G | 0.26 | 0.018 | 0.003 | 3.59E-10 |
| rs17554906 |  | 6 | 92226609 | C | G | 0.44 | 0.014 | 0.003 | 3.14E-08 |
| rs619087 |  | 6 | 94175279 | G | A | 0.42 | 0.014 | 0.003 | 3.10E-08 |
| rs6568832 |  | 6 | 97702876 | A | G | 0.75 | 0.019 | 0.003 | 1.74E-10 |
| rs12195240 |  | 6 | 98636905 | A | G | 0.29 | 0.025 | 0.003 | 1.08E-18 |
| rs6936160 |  | 6 | 100347745 | T | C | 0.70 | 0.020 | 0.003 | 4.20E-13 |
| rs12530388 |  | 6 | 101329173 | C | A | 0.51 | -0.018 | 0.003 | 5.83E-13 |
| rs3800227 |  | 6 | 108994161 | G | A | 0.74 | 0.017 | 0.003 | 3.64E-09 |
| rs118202 |  | 6 | 111658371 | T | G | 0.81 | -0.037 | 0.003 | 1.90E-29 |
| rs73008357 |  | 6 | 156431856 | C | A | 0.12 | -0.022 | 0.004 | 2.44E-08 |
| rs9331343 |  | 6 | 157738258 | C | T | 0.57 | -0.014 | 0.003 | 3.90E-08 |
| rs10698713 |  | 6 | 158882320 | A | G | 0.05 | -0.034 | 0.006 | 2.38E-09 |
| rs1737329 |  | 6 | 163807748 | G | C | 0.74 | 0.017 | 0.003 | 5.08E-09 |
| rs10272990 |  | 7 | 1703675 | C | T | 0.33 | -0.021 | 0.003 | 1.27E-14 |
| rs6948707 |  | 7 | 1870794 | G | T | 0.42 | 0.024 | 0.003 | 4.24E-21 |
| rs57257948 | rs10259715 | 7 | 3330595 | T | C | 0.21 | -0.019 | 0.003 | 6.42E-09 |
| rs13237637 |  | 7 | 3503207 | C | G | 0.49 | -0.024 | 0.003 | 1.54E-20 |
| rs79631993 |  | 7 | 69432311 | C | A | 0.22 | -0.017 | 0.003 | 3.67E-08 |
| rs7809303 |  | 7 | 69484366 | A | G | 0.33 | -0.021 | 0.003 | 3.48E-15 |
| rs7802996 |  | 7 | 77771983 | T | C | 0.17 | -0.021 | 0.003 | 1.06E-09 |
| rs1030015 |  | 7 | 78139581 | T | G | 0.52 | 0.014 | 0.003 | 2.15E-08 |
| rs4727189 |  | 7 | 88442568 | C | T | 0.34 | 0.015 | 0.003 | 3.00E-08 |
| rs76841737 |  | 7 | 91281409 | G | C | 0.10 | -0.023 | 0.004 | 3.26E-08 |
| rs11762736 | rs11768481 | 7 | 96630637 | G | A | 0.34 | -0.019 | 0.003 | 5.23E-12 |
| rs1799068 |  | 7 | 97707069 | T | G | 0.38 | 0.017 | 0.003 | 2.59E-10 |
| rs13437771 |  | 7 | 99071478 | G | A | 0.16 | -0.027 | 0.004 | 1.39E-14 |
| rs11766326 |  | 7 | 111100585 | C | T | 0.51 | -0.018 | 0.003 | 1.79E-11 |
| rs6968380 |  | 7 | 114940159 | A | G | 0.68 | -0.023 | 0.003 | 1.05E-17 |
| rs10233018 |  | 7 | 117523709 | G | A | 0.52 | 0.025 | 0.003 | 4.77E-22 |
| rs10953957 |  | 7 | 121954709 | A | G | 0.39 | 0.014 | 0.003 | 3.66E-08 |
| rs77283305 |  | 7 | 132593831 | A | G | 0.31 | -0.015 | 0.003 | 3.91E-08 |
| rs10279261 |  | 7 | 133589846 | A | G | 0.62 | -0.019 | 0.003 | 6.05E-13 |
| rs1561112 |  | 7 | 133840652 | C | T | 0.41 | -0.015 | 0.003 | 3.84E-09 |
| rs2952251 |  | 8 | 10143164 | G | A | 0.74 | 0.016 | 0.003 | 4.24E-08 |
| rs4326350 |  | 8 | 10763655 | G | C | 0.49 | -0.018 | 0.003 | 5.16E-12 |
| rs11780471 |  | 8 | 27344719 | A | G | 0.06 | -0.039 | 0.005 | 1.57E-13 |
| rs11783093 |  | 8 | 27425349 | T | C | 0.16 | -0.047 | 0.003 | 2.07E-41 |
| rs1565735 |  | 8 | 27426077 | A | T | 0.20 | -0.019 | 0.003 | 1.33E-09 |
| rs7836565 |  | 8 | 52569449 | T | C | 0.72 | -0.016 | 0.003 | 4.36E-08 |
| rs13261666 |  | 8 | 59814666 | T | G | 0.52 | -0.020 | 0.003 | 4.36E-15 |
| rs3850736 |  | 8 | 64912021 | G | C | 0.47 | 0.019 | 0.003 | 6.43E-14 |
| rs2063976 |  | 8 | 91096366 | T | C | 0.66 | -0.020 | 0.003 | 7.45E-14 |
| rs6993429 |  | 8 | 92733282 | A | C | 0.45 | -0.019 | 0.003 | 9.87E-14 |
| rs6986430 |  | 8 | 93048104 | C | T | 0.22 | -0.024 | 0.003 | 1.99E-15 |
| rs9987376 |  | 8 | 93190014 | G | T | 0.57 | -0.020 | 0.003 | 2.01E-15 |
| rs290601 |  | 8 | 115374642 | T | C | 0.27 | 0.016 | 0.003 | 1.14E-08 |
| rs3847244 |  | 9 | 3025368 | T | C | 0.47 | 0.019 | 0.003 | 2.60E-13 |
| rs11791671 |  | 9 | 3398679 | T | C | 0.07 | 0.028 | 0.005 | 4.24E-08 |
| rs7024924 |  | 9 | 8282399 | C | T | 0.17 | 0.019 | 0.003 | 1.90E-08 |
| rs6474609 |  | 9 | 10981069 | A | T | 0.59 | -0.016 | 0.003 | 1.71E-09 |
| rs1931431 |  | 9 | 11161799 | C | G | 0.48 | 0.018 | 0.003 | 8.56E-13 |
| rs7867822 |  | 9 | 20676454 | G | A | 0.67 | -0.015 | 0.003 | 2.76E-08 |
| rs10966092 |  | 9 | 23831658 | C | T | 0.27 | -0.020 | 0.003 | 1.12E-12 |
| rs10969352 |  | 9 | 29747488 | A | T | 0.50 | 0.014 | 0.003 | 1.82E-08 |
| rs4877285 |  | 9 | 81354129 | A | G | 0.67 | -0.018 | 0.003 | 2.10E-11 |
| rs1930371 |  | 9 | 81444104 | T | C | 0.24 | -0.017 | 0.003 | 7.09E-09 |
| rs2378662 |  | 9 | 86707289 | A | G | 0.54 | 0.015 | 0.003 | 2.67E-09 |
| rs1927901 |  | 9 | 120519111 | C | T | 0.55 | -0.014 | 0.003 | 3.10E-08 |
| rs4837631 |  | 9 | 122061948 | T | C | 0.45 | -0.015 | 0.003 | 2.03E-09 |
| rs1759433 |  | 9 | 128073097 | A | G | 0.48 | 0.015 | 0.003 | 1.69E-09 |
| rs34553878 |  | 9 | 134334588 | G | A | 0.11 | 0.025 | 0.004 | 1.17E-09 |
| rs7026534 |  | 9 | 134907263 | G | T | 0.70 | -0.017 | 0.003 | 2.68E-09 |
| rs10858334 |  | 9 | 137989785 | G | C | 0.14 | 0.023 | 0.004 | 1.18E-09 |
| rs10905461 |  | 10 | 8803551 | C | T | 0.75 | -0.016 | 0.003 | 2.36E-08 |
| rs7920501 |  | 10 | 10043159 | A | T | 0.47 | -0.016 | 0.003 | 1.25E-09 |
| rs1291821 |  | 10 | 11133823 | G | A | 0.53 | 0.014 | 0.003 | 1.39E-08 |
| rs11258417 |  | 10 | 13533053 | T | C | 0.39 | -0.015 | 0.003 | 2.71E-08 |
| rs7072776 |  | 10 | 22032942 | G | A | 0.71 | -0.022 | 0.003 | 5.66E-15 |
| rs2796793 |  | 10 | 36634124 | A | G | 0.45 | 0.014 | 0.003 | 1.55E-08 |
| rs1733760 |  | 10 | 56698174 | C | T | 0.51 | 0.015 | 0.003 | 6.70E-09 |
| rs7921378 |  | 10 | 63674885 | C | G | 0.48 | -0.023 | 0.003 | 6.10E-20 |
| rs7901883 |  | 10 | 103186838 | A | G | 0.23 | -0.019 | 0.003 | 1.98E-10 |
| rs11594623 |  | 10 | 103960351 | C | T | 0.23 | 0.027 | 0.003 | 7.45E-20 |
| rs11191269 |  | 10 | 104120522 | G | C | 0.19 | 0.018 | 0.003 | 4.61E-08 |
| rs28408682 |  | 10 | 104403310 | G | A | 0.60 | 0.017 | 0.003 | 1.41E-10 |
| rs12244388 |  | 10 | 104640052 | A | G | 0.35 | 0.026 | 0.003 | 4.31E-22 |
| rs943038 | rs111842178 | 10 | 104826261 | C | T | 0.23 | 0.022 | 0.003 | 2.24E-12 |
| rs34970111 |  | 10 | 106078937 | T | C | 0.46 | -0.015 | 0.003 | 1.28E-08 |
| rs9787523 |  | 10 | 106460460 | C | T | 0.42 | -0.016 | 0.003 | 1.42E-09 |
| rs11192347 |  | 10 | 106929313 | A | G | 0.10 | -0.026 | 0.004 | 6.15E-10 |
| rs10885480 |  | 10 | 115378364 | C | T | 0.28 | -0.019 | 0.003 | 3.83E-11 |
| rs4752018 |  | 10 | 118678712 | A | C | 0.23 | 0.019 | 0.003 | 4.42E-10 |
| rs9423279 |  | 10 | 125680419 | G | C | 0.65 | -0.019 | 0.003 | 3.06E-12 |
| rs6265 |  | 11 | 27679916 | T | C | 0.19 | -0.029 | 0.003 | 2.81E-19 |
| rs4275621 |  | 11 | 28652996 | G | A | 0.38 | -0.021 | 0.003 | 3.76E-16 |
| rs62618693 |  | 11 | 32956492 | T | C | 0.04 | -0.035 | 0.006 | 2.09E-08 |
| rs2939756 |  | 11 | 41436297 | A | G | 0.48 | -0.016 | 0.003 | 7.45E-10 |
| rs1381775 |  | 11 | 42442826 | C | T | 0.71 | -0.016 | 0.003 | 2.79E-08 |
| rs2959084 |  | 11 | 46078656 | A | G | 0.70 | 0.017 | 0.003 | 9.82E-10 |
| rs3740977 |  | 11 | 46393574 | C | T | 0.17 | 0.019 | 0.003 | 1.17E-08 |
| rs61886926 |  | 11 | 64133552 | T | C | 0.38 | -0.018 | 0.003 | 7.30E-12 |
| rs61884449 |  | 11 | 64485193 | T | C | 0.15 | 0.020 | 0.004 | 2.32E-08 |
| rs644740 |  | 11 | 65561468 | T | C | 0.46 | -0.014 | 0.003 | 3.67E-08 |
| rs7943721 |  | 11 | 73309393 | A | G | 0.83 | -0.021 | 0.003 | 3.58E-10 |
| rs7929518 |  | 11 | 85980958 | G | A | 0.77 | 0.019 | 0.003 | 2.55E-10 |
| rs586699 |  | 11 | 92289734 | A | G | 0.54 | -0.015 | 0.003 | 7.29E-09 |
| rs2155646 |  | 11 | 112912811 | C | T | 0.40 | 0.038 | 0.003 | 9.44E-48 |
| rs4471463 | rs78239456 | 11 | 112983595 | T | C | 0.38 | -0.018 | 0.003 | 9.37E-12 |
| rs1713676 |  | 11 | 113660576 | G | A | 0.52 | -0.017 | 0.003 | 5.38E-11 |
| rs238896 |  | 11 | 113994505 | A | G | 0.49 | -0.017 | 0.003 | 3.65E-11 |
| rs540860 |  | 11 | 121530888 | G | A | 0.54 | 0.018 | 0.003 | 5.75E-12 |
| rs1944689 |  | 11 | 121634334 | T | G | 0.79 | 0.018 | 0.003 | 1.27E-08 |
| rs1834306 |  | 11 | 122023187 | G | A | 0.58 | -0.014 | 0.003 | 1.96E-08 |
| rs1106363 |  | 11 | 131966264 | T | C | 0.34 | 0.017 | 0.003 | 9.20E-11 |
| rs2010921 |  | 11 | 132098205 | A | G | 0.31 | 0.017 | 0.003 | 2.47E-10 |
| rs11057005 |  | 12 | 16748721 | G | A | 0.44 | -0.016 | 0.003 | 9.12E-10 |
| rs13906 |  | 12 | 49952394 | T | C | 0.11 | -0.025 | 0.004 | 1.98E-09 |
| rs4759229 |  | 12 | 56474480 | G | A | 0.66 | 0.016 | 0.003 | 6.53E-09 |
| rs7969559 |  | 12 | 69655167 | G | A | 0.71 | -0.017 | 0.003 | 1.53E-09 |
| rs7134009 |  | 12 | 75263193 | C | T | 0.29 | -0.016 | 0.003 | 4.30E-08 |
| rs77215829 |  | 12 | 112618346 | C | A | 0.13 | -0.024 | 0.004 | 2.02E-10 |
| rs1109480 |  | 12 | 121083279 | A | G | 0.38 | -0.017 | 0.003 | 1.84E-10 |
| rs11611651 |  | 12 | 133380790 | A | G | 0.09 | 0.027 | 0.005 | 2.05E-09 |
| rs17197663 |  | 13 | 38172867 | A | G | 0.13 | -0.022 | 0.004 | 2.06E-08 |
| rs4264267 |  | 13 | 38359676 | T | C | 0.53 | 0.015 | 0.003 | 6.82E-09 |
| rs61959481 |  | 13 | 55834929 | A | G | 0.21 | -0.020 | 0.003 | 7.95E-11 |
| rs3098272 |  | 13 | 55931424 | C | A | 0.80 | -0.018 | 0.003 | 2.08E-08 |
| rs9538162 |  | 13 | 59265043 | C | T | 0.42 | 0.017 | 0.003 | 1.76E-11 |
| rs1413119 |  | 13 | 59339281 | T | C | 0.40 | -0.015 | 0.003 | 4.77E-09 |
| rs56367474 |  | 13 | 59454139 | T | C | 0.30 | -0.017 | 0.003 | 4.20E-10 |
| rs55786907 |  | 13 | 59871584 | G | A | 0.16 | 0.019 | 0.003 | 1.84E-08 |
| rs4886207 |  | 13 | 60705792 | C | T | 0.64 | -0.016 | 0.003 | 8.78E-10 |
| rs9540731 |  | 13 | 66949370 | T | C | 0.51 | -0.018 | 0.003 | 3.42E-12 |
| rs9545155 |  | 13 | 80191873 | C | T | 0.48 | -0.016 | 0.003 | 3.04E-10 |
| rs1772572 |  | 13 | 81191176 | A | C | 0.32 | -0.017 | 0.003 | 5.62E-10 |
| rs75674569 |  | 13 | 96823724 | A | G | 0.10 | -0.025 | 0.004 | 2.58E-09 |
| rs7333559 |  | 13 | 100546450 | A | G | 0.78 | -0.023 | 0.003 | 5.94E-14 |
| rs12855717 |  | 13 | 101252635 | T | C | 0.54 | 0.016 | 0.003 | 1.22E-09 |
| rs12878369 |  | 14 | 28346502 | A | C | 0.41 | 0.017 | 0.003 | 1.60E-11 |
| rs2145451 |  | 14 | 29316842 | C | T | 0.19 | -0.020 | 0.003 | 5.44E-10 |
| rs9323328 |  | 14 | 58653514 | G | A | 0.54 | -0.014 | 0.003 | 2.55E-08 |
| rs1811739 |  | 14 | 77529375 | A | G | 0.25 | 0.018 | 0.003 | 5.97E-10 |
| rs8005334 |  | 14 | 79563654 | G | T | 0.36 | 0.017 | 0.003 | 3.44E-10 |
| rs34940743 |  | 14 | 80102233 | G | A | 0.35 | 0.016 | 0.003 | 2.80E-09 |
| rs2925128 |  | 14 | 98362355 | T | C | 0.39 | 0.017 | 0.003 | 3.67E-10 |
| rs1381287 |  | 14 | 98597552 | T | C | 0.47 | 0.018 | 0.003 | 1.81E-12 |
| rs55913542 |  | 14 | 99693843 | T | G | 0.18 | 0.019 | 0.003 | 3.25E-08 |
| rs1435672 |  | 15 | 36399479 | C | T | 0.56 | 0.014 | 0.003 | 3.82E-08 |
| rs281296 |  | 15 | 47685010 | A | G | 0.36 | 0.025 | 0.003 | 1.59E-20 |
| rs1435741 |  | 15 | 47935843 | A | G | 0.43 | 0.018 | 0.003 | 1.09E-12 |
| rs56902655 |  | 15 | 63898709 | G | T | 0.14 | -0.022 | 0.004 | 4.09E-09 |
| rs2289791 |  | 15 | 67476952 | T | G | 0.25 | -0.018 | 0.003 | 2.01E-09 |
| rs60833441 |  | 15 | 74048768 | G | A | 0.46 | -0.014 | 0.003 | 2.28E-08 |
| rs62007780 |  | 15 | 78025464 | T | G | 0.42 | -0.016 | 0.003 | 7.48E-10 |
| rs12442563 |  | 15 | 83893243 | T | G | 0.22 | -0.023 | 0.003 | 3.13E-14 |
| rs4310804 |  | 15 | 96858409 | G | C | 0.25 | -0.018 | 0.003 | 7.55E-10 |
| rs8027457 |  | 15 | 99204101 | C | T | 0.51 | 0.015 | 0.003 | 1.88E-09 |
| rs1139897 |  | 16 | 720986 | A | G | 0.23 | -0.024 | 0.003 | 1.77E-15 |
| rs11076962 |  | 16 | 5811367 | C | T | 0.28 | 0.018 | 0.003 | 1.20E-10 |
| rs7192140 |  | 16 | 10173748 | C | T | 0.50 | -0.017 | 0.003 | 3.40E-11 |
| rs9922607 |  | 16 | 17570220 | T | C | 0.20 | -0.022 | 0.003 | 3.42E-12 |
| rs9941217 |  | 16 | 18050926 | G | C | 0.35 | -0.019 | 0.003 | 3.50E-12 |
| rs7188873 |  | 16 | 24727064 | G | A | 0.61 | 0.020 | 0.003 | 8.46E-15 |
| rs6497840 |  | 16 | 25351633 | A | G | 0.71 | 0.023 | 0.003 | 2.01E-15 |
| rs4785187 |  | 16 | 49766772 | A | G | 0.22 | 0.020 | 0.003 | 6.55E-11 |
| rs8050598 |  | 16 | 49891964 | T | C | 0.25 | 0.019 | 0.003 | 1.76E-10 |
| rs12918191 |  | 16 | 50945156 | G | A | 0.24 | -0.020 | 0.003 | 3.14E-11 |
| rs9302604 |  | 16 | 69576894 | G | A | 0.44 | 0.019 | 0.003 | 3.29E-13 |
| rs9936784 |  | 16 | 72230694 | G | T | 0.53 | 0.014 | 0.003 | 4.33E-08 |
| rs62052916 |  | 16 | 72574550 | T | A | 0.07 | -0.032 | 0.005 | 1.62E-10 |
| rs4788676 |  | 16 | 72950468 | C | T | 0.23 | -0.018 | 0.003 | 4.92E-09 |
| rs4888437 | rs61537885 | 16 | 75618058 | A | C | 0.04 | -0.040 | 0.007 | 8.06E-09 |
| rs117657830 |  | 16 | 75766873 | G | A | 0.04 | -0.038 | 0.006 | 3.18E-09 |
| rs1050847 |  | 16 | 87443734 | T | C | 0.56 | -0.015 | 0.003 | 7.37E-09 |
| rs11642231 |  | 16 | 89608702 | A | G | 0.37 | -0.016 | 0.003 | 3.44E-09 |
| rs4790874 |  | 17 | 1995177 | T | C | 0.53 | 0.017 | 0.003 | 8.43E-12 |
| rs11078713 |  | 17 | 7795972 | G | A | 0.42 | -0.015 | 0.003 | 1.59E-08 |
| rs28441558 |  | 17 | 7803118 | C | T | 0.06 | -0.036 | 0.006 | 1.24E-10 |
| rs11651955 |  | 17 | 16235462 | A | G | 0.50 | -0.014 | 0.003 | 3.74E-08 |
| rs67777803 |  | 17 | 27323322 | T | G | 0.17 | -0.025 | 0.003 | 3.18E-13 |
| rs2344976 |  | 17 | 30685935 | C | T | 0.61 | -0.015 | 0.003 | 7.98E-09 |
| rs72836318 |  | 17 | 44121579 | C | T | 0.25 | -0.017 | 0.003 | 7.00E-09 |
| rs17692129 |  | 17 | 44793283 | T | C | 0.33 | 0.020 | 0.003 | 4.57E-13 |
| rs75919030 |  | 17 | 50193197 | C | T | 0.27 | -0.021 | 0.003 | 3.35E-13 |
| rs2938134 |  | 17 | 50243397 | A | C | 0.67 | -0.018 | 0.003 | 3.14E-10 |
| rs2587507 |  | 17 | 77790135 | C | T | 0.50 | -0.015 | 0.003 | 8.69E-09 |
| rs34342129 |  | 18 | 5872472 | C | T | 0.51 | -0.014 | 0.003 | 2.13E-08 |
| rs4476253 |  | 18 | 25253297 | A | G | 0.24 | -0.018 | 0.003 | 5.78E-10 |
| rs7505855 |  | 18 | 31696075 | T | C | 0.59 | -0.017 | 0.003 | 5.31E-11 |
| rs8096225 |  | 18 | 36921851 | C | A | 0.70 | 0.016 | 0.003 | 2.63E-08 |
| rs67050670 |  | 18 | 39297254 | G | A | 0.23 | -0.020 | 0.003 | 2.34E-11 |
| rs12607929 | rs2359180 | 18 | 41314562 | G | T | 0.37 | -0.014 | 0.003 | 4.98E-08 |
| rs72898831 |  | 18 | 42658643 | G | A | 0.16 | -0.024 | 0.004 | 4.14E-12 |
| rs8083764 |  | 18 | 49874515 | T | G | 0.31 | -0.016 | 0.003 | 7.97E-09 |
| rs1373178 |  | 18 | 49967811 | G | T | 0.59 | -0.020 | 0.003 | 4.16E-15 |
| rs62098013 |  | 18 | 50863861 | A | G | 0.37 | 0.018 | 0.003 | 2.24E-11 |
| rs72938304 |  | 18 | 53661743 | A | G | 0.11 | -0.027 | 0.004 | 1.36E-11 |
| rs11872397 |  | 18 | 72535282 | A | G | 0.25 | -0.017 | 0.003 | 5.20E-09 |
| rs71367544 |  | 18 | 77574374 | T | C | 0.20 | 0.021 | 0.003 | 8.54E-11 |
| rs76608582 |  | 19 | 4474725 | A | C | 0.05 | -0.035 | 0.006 | 4.88E-09 |
| rs10853981 |  | 19 | 4965064 | A | G | 0.33 | 0.015 | 0.003 | 4.88E-08 |
| rs113230003 |  | 19 | 18460956 | A | G | 0.26 | -0.019 | 0.003 | 1.05E-10 |
| rs8103660 |  | 19 | 18566395 | C | T | 0.35 | 0.016 | 0.003 | 3.03E-09 |
| rs117734003 |  | 19 | 51129745 | C | G | 0.07 | 0.030 | 0.005 | 2.57E-09 |
| rs1126757 |  | 19 | 55879872 | T | C | 0.47 | 0.014 | 0.003 | 2.92E-08 |
| rs6050446 |  | 20 | 25195509 | G | A | 0.97 | 0.054 | 0.008 | 8.80E-13 |
| rs6058782 |  | 20 | 29946968 | T | C | 0.91 | 0.030 | 0.004 | 1.78E-11 |
| rs1555445 |  | 20 | 31175258 | T | A | 0.32 | 0.019 | 0.003 | 7.75E-12 |
| rs6073075 |  | 20 | 42015801 | A | T | 0.82 | -0.019 | 0.003 | 2.44E-08 |
| rs910912 |  | 20 | 54462393 | C | T | 0.74 | -0.017 | 0.003 | 7.82E-09 |
| rs6011779 |  | 20 | 61984317 | T | C | 0.81 | -0.019 | 0.003 | 2.83E-09 |
| rs3810496 |  | 20 | 62406886 | C | T | 0.62 | 0.016 | 0.003 | 1.54E-09 |
| rs4818005 |  | 21 | 40588819 | A | G | 0.58 | -0.020 | 0.003 | 1.09E-14 |
| rs139896 |  | 22 | 38397797 | C | T | 0.65 | 0.015 | 0.003 | 7.14E-09 |
| rs4822102 |  | 22 | 42698430 | T | C | 0.62 | -0.017 | 0.003 | 2.78E-10 |
| rs9627272 |  | 22 | 46442288 | C | G | 0.41 | -0.015 | 0.003 | 2.42E-09 |

SI Table B: Two-sample Mendelian Randomization estimates of the association between smoking initiation excluding SNPs within the MHC and incidence of multiple sclerosis.

A two sample MR analysis was undertaken to obtain causal estimates of genetically predicted smoking initiation, excluding SNPs within the MHC, on MS susceptibility. MR and sensitivity analyses were performed using the TwoSampleMR R package with a comparison across five different methods. Odds ratios are expressed per unit increase in log odds of ever smoking regularly (smoking initiation), with a one SD increase in genetically predicted smoking initiation corresponding to a 10% increased risk of smoking. The genetic variants used to proxy smoking initiation are the conditionally independent genome-wide significant SNPs taken from the GWAS & Sequencing Consortium of Alcohol and Nicotine use (GSCAN) consortium detailed in Table A in S1 Data excluding SNPs within the MHC (defined as base positions 24,000,000 to 35,000,000 on chromosome 6 [GRCh37]). The estimates of their association with MS are taken from the 2019 MS Chip International Multiple Sclerosis Genetics Consortium (IMSGC) meta-analysis. CI: Confidence Interval; SE: Standard Error.

| **Method** | **Odds Ratio** | **Lower CI** | **Upper CI** | **P value** |
| --- | --- | --- | --- | --- |
| Inverse variance weighted | 1.06 | 0.95 | 1.19 | 0.30 |
| Weighted median | 1.09 | 0.94 | 1.26 | 0.24 |
| Weighted mode | 1.15 | 0.79 | 1.67 | 0.48 |
| MR Egger | 1.06 | 0.64 | 1.74 | 0.83 |
| Robust adjusted profile score (RAPS) | 1.06 | 0.95 | 1.19 | 0.30 |

SI Table C: List of independent SNPs associated with lifetime smoking at the genome-wide level of significance (p<5x10-8) used as instruments in the Mendelian randomization analysis.

Genetic variants and effect sizes from GWAS of lifetime smoking^2^

SNP: Single Nucleotide Polymorphism; CHR: Chromosome; BP: Base pair position of the variant according to human reference sequence (GRCh37), Hg19; EA: Effect Allele; OA: Other Allele; EAF: Effect Allele Frequency; beta per effect allele; Standard error of the beta; p value: Strength of evidence against the null hypothesis of no association between variant and outcome.

| **SNP** | **Original SNP if proxy used in analysis (correlation)** | **CHR** | **Base pair**  **Position** | **EA** | **OA** | **EAF** | **beta** | **Stan-**  **dard**  **Error** | **p value** |
| --- | --- | --- | --- | --- | --- | --- | --- | --- | --- |
| rs11210229 |  | 1 | 73860028 | A | G | 0.38 | 0.017 | 0.002 | 2.00E-16 |
| rs549845 |  | 1 | 44076469 | G | A | 0.30 | 0.016 | 0.002 | 8.30E-14 |
| rs10922907 |  | 1 | 91193049 | A | T | 0.45 | 0.015 | 0.002 | 3.00E-13 |
| rs7553348 |  | 1 | 75005067 | G | A | 0.44 | 0.014 | 0.002 | 5.20E-12 |
| rs7528604 |  | 1 | 66407352 | G | A | 0.57 | 0.014 | 0.002 | 5.70E-12 |
| rs1933270 |  | 1 | 49977965 | T | G | 0.36 | 0.013 | 0.002 | 1.50E-10 |
| rs7519626 |  | 1 | 99514554 | C | T | 0.32 | 0.012 | 0.002 | 1.20E-08 |
| rs9435340 |  | 1 | 107593201 | T | A | 0.34 | 0.012 | 0.002 | 1.20E-08 |
| rs4949465 |  | 1 | 32178489 | T | C | 0.87 | -0.017 | 0.003 | 1.70E-08 |
| rs10918701 |  | 1 | 162090536 | G | A | 0.37 | 0.012 | 0.002 | 2.10E-08 |
| rs1193237 |  | 1 | 7526486 | G | C | 0.44 | -0.011 | 0.002 | 2.80E-08 |
| rs1931263 |  | 1 | 96175101 | G | T | 0.51 | -0.011 | 0.002 | 4.00E-08 |
| rs2890772 |  | 2 | 146175106 | G | T | 0.41 | -0.020 | 0.002 | 2.10E-22 |
| rs62155874 |  | 2 | 105973094 | A | G | 0.87 | -0.024 | 0.003 | 5.20E-16 |
| rs2867112 |  | 2 | 651349 | T | G | 0.83 | 0.021 | 0.003 | 4.80E-15 |
| rs7569203 |  | 2 | 45154418 | A | C | 0.69 | -0.016 | 0.002 | 7.40E-13 |
| rs12623702 |  | 2 | 202885506 | A | G | 0.61 | -0.014 | 0.002 | 7.70E-12 |
| rs4671357 |  | 2 | 60136176 | T | C | 0.52 | -0.014 | 0.002 | 1.10E-11 |
| rs4473348 |  | 2 | 182073742 | A | T | 0.25 | -0.015 | 0.002 | 6.40E-11 |
| rs2678670 |  | 2 | 104469564 | A | T | 0.49 | 0.013 | 0.002 | 3.10E-10 |
| rs62135536 |  | 2 | 44326028 | C | T | 0.97 | 0.035 | 0.006 | 8.00E-10 |
| rs3811038 |  | 2 | 113240183 | T | C | 0.72 | -0.014 | 0.002 | 8.90E-10 |
| rs359243 |  | 2 | 60475509 | T | C | 0.39 | -0.013 | 0.002 | 9.50E-10 |
| rs13016665 |  | 2 | 57995348 | C | A | 0.58 | -0.012 | 0.002 | 1.80E-09 |
| rs3769949 |  | 2 | 166199284 | T | A | 0.53 | -0.012 | 0.002 | 2.50E-09 |
| rs13009008 |  | 2 | 174043233 | A | G | 0.33 | 0.012 | 0.002 | 4.60E-09 |
| rs6741228 |  | 2 | 22548774 | T | C | 0.43 | 0.011 | 0.002 | 1.60E-08 |
| rs62175972 |  | 2 | 161362830 | T | C | 0.97 | 0.031 | 0.006 | 1.70E-08 |
| rs6778080 |  | 3 | 49317338 | T | C | 0.27 | 0.016 | 0.002 | 1.30E-12 |
| rs326341 |  | 3 | 107811142 | G | A | 0.52 | 0.014 | 0.002 | 1.20E-11 |
| rs421983 |  | 3 | 84892866 | T | C | 0.52 | 0.013 | 0.002 | 3.30E-10 |
| rs6779302 |  | 3 | 16859710 | G | T | 0.63 | -0.013 | 0.002 | 1.20E-09 |
| rs9842947 |  | 3 | 157412246 | C | T | 0.33 | -0.013 | 0.002 | 3.10E-09 |
| rs775758 |  | 3 | 77582005 | A | T | 0.43 | 0.012 | 0.002 | 1.10E-08 |
| rs73220544 |  | 3 | 131074511 | A | C | 0.84 | -0.016 | 0.003 | 1.50E-08 |
| rs17576594 |  | 4 | 147952241 | G | A | 0.72 | 0.016 | 0.002 | 1.70E-12 |
| rs72678864 |  | 4 | 112422145 | G | A | 0.83 | 0.018 | 0.003 | 1.60E-11 |
| rs317021 |  | 4 | 35418368 | T | A | 0.81 | -0.017 | 0.003 | 1.10E-10 |
| rs624833 |  | 4 | 2881256 | T | G | 0.69 | 0.013 | 0.002 | 6.60E-10 |
| rs61796681 |  | 4 | 23678196 | A | T | 0.91 | -0.019 | 0.004 | 4.20E-08 |
| rs986391 |  | 5 | 166993972 | G | A | 0.37 | 0.016 | 0.002 | 9.40E-15 |
| rs329120 |  | 5 | 133861756 | C | T | 0.58 | 0.014 | 0.002 | 6.30E-12 |
| rs13153393 |  | 5 | 167604213 | A | G | 0.88 | -0.020 | 0.003 | 2.50E-10 |
| rs11948770 |  | 5 | 13246336 | T | C | 0.77 | -0.015 | 0.002 | 4.90E-10 |
| rs71627581 |  | 5 | 43161351 | G | A | 0.89 | 0.019 | 0.003 | 1.60E-09 |
| rs10052591 |  | 5 | 50812738 | T | C | 0.57 | 0.012 | 0.002 | 2.10E-09 |
| rs4957528 |  | 5 | 106420589 | A | C | 0.21 | -0.015 | 0.002 | 4.20E-09 |
| rs245774 |  | 5 | 170530930 | A | G | 0.27 | -0.013 | 0.002 | 7.40E-09 |
| rs4571506 |  | 5 | 87756918 | C | T | 0.54 | 0.011 | 0.002 | 1.50E-08 |
| rs2080870 |  | 5 | 60388313 | A | T | 0.26 | 0.012 | 0.002 | 4.90E-08 |
| rs7766610 |  | 6 | 111707821 | C | A | 0.18 | 0.018 | 0.003 | 2.20E-12 |
| rs6935954 |  | 6 | 26255451 | A | G | 0.42 | 0.014 | 0.002 | 8.20E-12 |
| rs12202536 |  | 6 | 67475273 | A | G | 0.51 | -0.012 | 0.002 | 2.80E-09 |
| rs2894808 |  | 6 | 52861990 | T | A | 0.92 | -0.022 | 0.004 | 3.50E-09 |
| rs2254710 |  | 6 | 37477000 | C | A | 0.24 | 0.013 | 0.002 | 3.50E-08 |
| rs10226228 |  | 7 | 32315613 | A | G | 0.63 | -0.016 | 0.002 | 2.00E-15 |
| rs2401924 |  | 7 | 115057862 | G | C | 0.50 | 0.015 | 0.002 | 2.70E-14 |
| rs7807019 |  | 7 | 117543063 | A | G | 0.54 | -0.015 | 0.002 | 6.70E-14 |
| rs1922018 |  | 7 | 3560401 | C | T | 0.36 | 0.014 | 0.002 | 3.00E-12 |
| rs10282292 |  | 7 | 111092478 | C | T | 0.36 | 0.013 | 0.002 | 5.90E-10 |
| rs11762736 | rs11768481 | 7 | 96630637 | A | G | 0.67 | 0.013 | 0.002 | 9.90E-10 |
| rs6962772 |  | 7 | 99081730 | A | G | 0.85 | 0.016 | 0.003 | 7.80E-09 |
| rs4731925 |  | 7 | 132664757 | C | T | 0.32 | -0.012 | 0.002 | 2.60E-08 |
| rs6957896 |  | 7 | 132309592 | C | T | 0.50 | -0.011 | 0.002 | 4.50E-08 |
| rs11783093 |  | 8 | 27425349 | C | T | 0.84 | 0.023 | 0.003 | 1.20E-16 |
| rs35169606 |  | 8 | 9604066 | T | G | 0.61 | 0.013 | 0.002 | 1.20E-09 |
| rs2062882 |  | 8 | 91839576 | G | A | 0.59 | -0.012 | 0.002 | 1.10E-08 |
| rs72674867 |  | 8 | 95578201 | A | T | 0.76 | 0.013 | 0.002 | 3.80E-08 |
| rs3025327 | rs113382419 | 9 | 136467344 | G | C | 0.89 | -0.041 | 0.003 | 3.00E-37 |
| rs13296519 |  | 9 | 128471924 | G | T | 0.61 | -0.014 | 0.002 | 8.10E-12 |
| rs1221148 |  | 9 | 122046875 | C | G | 0.59 | 0.013 | 0.002 | 7.30E-11 |
| rs4543592 |  | 9 | 3014254 | T | C | 0.52 | -0.012 | 0.002 | 4.50E-10 |
| rs7039819 |  | 9 | 82430418 | G | A | 0.43 | 0.013 | 0.002 | 5.10E-10 |
| rs1246265 |  | 9 | 86761745 | T | C | 0.30 | -0.013 | 0.002 | 4.20E-09 |
| rs12244388 |  | 10 | 104640052 | G | A | 0.66 | -0.019 | 0.002 | 1.40E-19 |
| rs3896224 |  | 10 | 106467853 | A | G | 0.59 | 0.014 | 0.002 | 1.10E-11 |
| rs11255908 |  | 10 | 8802912 | T | G | 0.74 | -0.015 | 0.002 | 2.30E-10 |
| rs2675638 |  | 10 | 63576286 | G | A | 0.58 | 0.012 | 0.002 | 1.30E-09 |
| rs7077678 |  | 10 | 104438565 | C | T | 0.62 | 0.012 | 0.002 | 2.60E-09 |
| rs17553262 |  | 10 | 92912773 | A | C | 0.88 | -0.018 | 0.003 | 5.30E-09 |
| rs10823968 |  | 10 | 74738269 | A | T | 0.63 | 0.012 | 0.002 | 2.10E-08 |
| rs9919670 |  | 11 | 112877304 | G | A | 0.61 | -0.022 | 0.002 | 7.60E-27 |
| rs17309874 |  | 11 | 27667236 | G | A | 0.74 | -0.016 | 0.002 | 9.70E-13 |
| rs4391802 |  | 11 | 28674592 | A | G | 0.71 | 0.015 | 0.002 | 1.40E-11 |
| rs112282219 |  | 11 | 46632809 | G | A | 0.96 | -0.033 | 0.005 | 3.80E-11 |
| rs75742406 |  | 11 | 17070365 | G | A | 0.74 | 0.014 | 0.002 | 1.30E-09 |
| rs34866095 |  | 11 | 16377356 | A | G | 0.69 | -0.012 | 0.002 | 1.20E-08 |
| rs10879871 |  | 12 | 75380511 | T | G | 0.34 | -0.014 | 0.002 | 5.00E-11 |
| rs7297175 |  | 12 | 56473808 | T | C | 0.43 | -0.012 | 0.002 | 6.60E-09 |
| rs12831617 |  | 12 | 84758368 | C | T | 0.76 | -0.013 | 0.002 | 1.90E-08 |
| rs74086911 |  | 12 | 50015942 | G | A | 0.93 | 0.021 | 0.004 | 2.10E-08 |
| rs7333559 |  | 13 | 100546450 | G | A | 0.21 | 0.015 | 0.002 | 3.20E-10 |
| rs6562474 |  | 13 | 67332812 | C | G | 0.65 | 0.012 | 0.002 | 1.00E-08 |
| rs3742365 |  | 14 | 104198251 | T | C | 0.60 | -0.016 | 0.002 | 2.50E-14 |
| rs7155595 |  | 14 | 77502546 | A | C | 0.67 | -0.013 | 0.002 | 2.50E-09 |
| rs860326 |  | 14 | 57342912 | C | T | 0.43 | 0.012 | 0.002 | 2.70E-09 |
| rs8042849 |  | 15 | 78817929 | C | T | 0.34 | 0.028 | 0.002 | 1.80E-39 |
| rs35175834 |  | 15 | 47680815 | G | A | 0.79 | -0.024 | 0.002 | 4.60E-22 |
| rs8042134 |  | 15 | 97514404 | T | G | 0.54 | -0.014 | 0.002 | 1.30E-12 |
| rs6598539 |  | 15 | 99204483 | T | C | 0.49 | -0.012 | 0.002 | 4.50E-09 |
| rs28485305 |  | 15 | 74044197 | C | T | 0.63 | 0.012 | 0.002 | 2.60E-08 |
| rs889398 |  | 16 | 69556715 | C | T | 0.59 | 0.013 | 0.002 | 6.30E-11 |
| rs369230 |  | 16 | 89645437 | G | T | 0.31 | -0.013 | 0.002 | 1.80E-09 |
| rs12708665 |  | 16 | 24728227 | A | G | 0.28 | -0.013 | 0.002 | 3.50E-09 |
| rs1050847 |  | 16 | 87443734 | C | T | 0.43 | 0.011 | 0.002 | 1.40E-08 |
| rs11861214 |  | 16 | 746611 | G | T | 0.78 | 0.014 | 0.002 | 2.00E-08 |
| rs60952428 |  | 16 | 75640521 | T | C | 0.91 | 0.019 | 0.003 | 3.00E-08 |
| rs57611503 |  | 16 | 31165795 | G | A | 0.48 | 0.011 | 0.002 | 4.00E-08 |
| rs8614 |  | 17 | 27588806 | C | A | 0.82 | -0.017 | 0.003 | 1.80E-10 |
| rs67596067 |  | 17 | 50333733 | G | A | 0.65 | -0.013 | 0.002 | 1.20E-09 |
| rs732083 |  | 17 | 37834367 | G | A | 0.33 | 0.012 | 0.002 | 1.50E-08 |
| rs9904288 |  | 17 | 47031973 | T | C | 0.71 | 0.012 | 0.002 | 3.10E-08 |
| rs71367545 |  | 18 | 77576337 | G | A | 0.79 | -0.015 | 0.002 | 1.40E-09 |
| rs62098013 |  | 18 | 50863861 | G | A | 0.64 | -0.012 | 0.002 | 4.10E-09 |
| rs12967855 |  | 18 | 35138245 | A | G | 0.33 | 0.012 | 0.002 | 3.10E-08 |
| rs76608582 |  | 19 | 4474725 | C | A | 0.95 | 0.031 | 0.005 | 3.20E-10 |
| rs35343344 |  | 19 | 18471610 | C | A | 0.73 | 0.013 | 0.002 | 8.80E-09 |
| rs6011779 |  | 20 | 61984317 | C | T | 0.19 | 0.028 | 0.003 | 2.30E-27 |
| rs6119897 |  | 20 | 31145415 | G | A | 0.76 | -0.018 | 0.002 | 3.60E-15 |
| rs4814873 |  | 20 | 19616429 | C | T | 0.77 | 0.014 | 0.002 | 2.90E-09 |
| rs12481282 |  | 20 | 44761377 | G | C | 0.72 | -0.013 | 0.002 | 7.80E-09 |
| rs348809 |  | 20 | 59032097 | A | G | 0.35 | -0.012 | 0.002 | 1.30E-08 |
| rs2838834 |  | 21 | 46665208 | C | T | 0.70 | -0.013 | 0.002 | 6.30E-10 |
| rs147412694 |  | 21 | 40702786 | G | A | 0.85 | -0.017 | 0.003 | 2.90E-09 |
| rs202645 |  | 22 | 41798520 | A | G | 0.20 | -0.015 | 0.002 | 3.90E-09 |
| rs136233 |  | 22 | 31212410 | A | G | 0.81 | -0.014 | 0.003 | 1.80E-08 |

SI Table D: Two-sample Mendelian Randomization estimates of the association between lifetime smoking excluding SNPs within the MHC and incidence of multiple sclerosis.

A two sample MR analysis was undertaken to obtain causal estimates of genetically predicted lifetime smoking, excluding SNPs within the MHC, on MS susceptibility. MR and sensitivity analyses were performed using the TwoSampleMR R package with a comparison across five different methods. Odds ratios are expressed per one standard deviation increase of the lifetime smoking index. A standard deviation increase in the lifetime smoking score is equivalent to an individual smoking 20 cigarettes a day for 15 years and stopping 17 years ago or an individual smoking 60 cigarettes a day for 13 years and stopping 22 years ago. The genetic variants used to proxy lifetime smoking are the independent genome-wide significant SNPs taken from the GWAS of lifetime smoking performed by Wootton et al. 2019 detailed in Table C in S1 Data excluding SNPs within the MHC (defined as base positions 24,000,000 to 35,000,000 on chromosome 6 [GRCh37]). The estimates of their association with MS are taken from the 2019 MS Chip International Multiple Sclerosis Genetics Consortium (IMSGC) meta-analysis. CI: Confidence Interval; SE: Standard Error.

| **Method** | **Odds Ratio** | **Lower CI** | **Upper CI** | **SE** | **P value** |
| --- | --- | --- | --- | --- | --- |
| Inverse variance weighted | 1.10 | 0.86 | 1.40 | 0.12 | 0.46 |
| Weighted median | 1.05 | 0.76 | 1.44 | 0.16 | 0.78 |
| Weighted mode | 1.05 | 0.49 | 2.23 | 0.39 | 0.91 |
| MR Egger | 1.35 | 0.49 | 3.67 | 0.51 | 0.56 |
| Robust adjusted profile score (RAPS) | 1.12 | 0.88 | 1.43 | 0.12 | 0.35 |

SI Table E: Two-sample Mendelian Randomization estimates of the association between incidence of multiple sclerosis excluding SNPs within the MHC and smoking initiation.

A two sample MR analysis was undertaken to obtain causal estimates of genetically predicted incidence of multiple sclerosis on smoking initiation, excluding SNPs within the MHC. MR and sensitivity analyses were performed using the TwoSampleMR R package with a comparison across five different methods. Odds ratios are expressed per unit increase in log odds of incidence of MS. The genetic variants used to proxy Multiple Sclerosis are the independent genome-wide significant SNPs excluding those within the MHC (defined as base positions 24,000,000 to 35,000,000 on chromosome 6 [GRCh37]) taken from the 2019 MS Chip International Multiple Sclerosis Genetics Consortium (IMSGC) meta-analysis. The estimates of their association with smoking initiation are taken from the GWAS & Sequencing Consortium of Alcohol and Nicotine use (GSCAN) consortium. CI: Confidence Interval; SE: Standard Error.

| **Method** | **Odds Ratio** | **Lower CI** | **Upper CI** | **SE** | **P value** |
| --- | --- | --- | --- | --- | --- |
| Inverse variance weighted | 0.999 | 0.992 | 1.007 | 0.004 | 0.891 |
| Weighted median | 1.004 | 0.994 | 1.013 | 0.005 | 0.464 |
| Weighted mode | 1.012 | 0.988 | 1.037 | 0.012 | 0.339 |
| MR Egger | 1.003 | 0.979 | 1.028 | 0.013 | 0.810 |
| Robust adjusted profile score (RAPS) | 1.002 | 0.994 | 1.010 | 0.004 | 0.619 |

SI Table F: Two-sample Mendelian Randomization estimates of the association between incidence of

multiple sclerosis excluding SNPs within the MHC and lifetime smoking.

A two sample MR analysis was undertaken to obtain causal estimates of genetically predicted incidence of multiple sclerosis on lifetime smoking, excluding SNPs within the MHC. MR and sensitivity analyses were performed using the TwoSampleMR R package with a comparison across five different methods. Odds ratios for lifetime smoking are expressed per unit increase in log odds of incidence of MS. The genetic variants used to proxy Multiple Sclerosis are the independent genome-wide significant SNPs excluding those within the MHC (defined as base positions 24,000,000 to 35,000,000 on chromosome 6 [GRCh37]) taken from the 2019 MS Chip International Multiple Sclerosis Genetics Consortium (IMSGC) meta-analysis. The estimates of their association with lifetime smoking are taken from the GWAS of lifetime smoking performed by Wootton et al. 2019. CI: Confidence Interval; SE: Standard Error.

| **Method** | **Odds Ratio** | **Lower CI** | **Upper CI** | **SE** | **P value** |
| --- | --- | --- | --- | --- | --- |
| Inverse variance weighted | 1.001 | 0.997 | 1.004 | 0.002 | 0.716 |
| Weighted median | 1.000 | 0.996 | 1.003 | 0.002 | 0.794 |
| Weighted mode | 0.996 | 0.988 | 1.005 | 0.004 | 0.382 |
| MR Egger | 1.001 | 0.991 | 1.011 | 0.005 | 0.864 |
| Robust adjusted profile score (RAPS) | 1.000 | 0.997 | 1.003 | 0.002 | 0.972 |

SI Table G: Two-sample Mendelian Randomization estimates of the association between incidence of multiple sclerosis of SNPs within the MHC and smoking initiation.

A two sample MR analysis was undertaken to obtain causal estimates of genetically predicted incidence of multiple sclerosis on smoking initiation, using SNPs within the MHC. MR and sensitivity analyses were performed using the TwoSampleMR R package with a comparison across five different methods. Odds ratios are expressed per unit increase in log odds of incidence of MS. The genetic variants used to proxy Multiple Sclerosis are the independent genome-wide significant SNPs within the MHC (defined as base positions 24,000,000 to 35,000,000 on chromosome 6 [GRCh37]) taken from the 2019 MS Chip International Multiple Sclerosis Genetics Consortium (IMSGC) meta-analysis. The estimates of their association with smoking initiation are taken from the GWAS & Sequencing Consortium of Alcohol and Nicotine use (GSCAN) consortium. CI: Confidence Interval; SE: Standard Error.

| **Method** | **Odds Ratio** | **Lower CI** | **Upper CI** | **SE** | **P value** |
| --- | --- | --- | --- | --- | --- |
| Inverse variance weighted | 0.989 | 0.972 | 1.007 | 0.009 | 0.225 |
| Weighted median | 0.988 | 0.970 | 1.006 | 0.009 | 0.195 |
| Weighted mode | 0.989 | 0.971 | 1.008 | 0.010 | 0.261 |
| MR Egger | 0.993 | 0.957 | 1.030 | 0.019 | 0.718 |
| Robust adjusted profile score (RAPS) | 0.991 | 0.978 | 1.004 | 0.007 | 0.162 |

SI Table H: Two-sample Mendelian Randomization estimates of the association between incidence of multiple sclerosis of SNPs within the MHC and lifetime smoking.

A two sample MR analysis was undertaken to obtain causal estimates of genetically predicted incidence of multiple sclerosis on lifetime smoking, using SNPs within the MHC. MR and sensitivity analyses were performed using the TwoSampleMR R package with a comparison across five different methods. Odds ratios for lifetime smoking are expressed per unit increase in log odds of incidence of MS. The genetic variants used to proxy Multiple Sclerosis are the independent genome-wide significant SNPs within the MHC (defined as base positions 24,000,000 to 35,000,000 on chromosome 6 [GRCh37]) taken from the 2019 MS Chip International Multiple Sclerosis Genetics Consortium (IMSGC) meta-analysis. The estimates of their association with lifetime smoking are taken from the GWAS of lifetime smoking performed by Wootton et al. 2019. CI: Confidence Interval; SE: Standard Error.

| **Method** | **Beta** | **Lower CI** | **Upper CI** | **SE** | **P value** |
| --- | --- | --- | --- | --- | --- |
| Inverse variance weighted | 1.001 | 0.993 | 1.010 | 0.004 | 0.756 |
| Weighted median | 0.998 | 0.991 | 1.006 | 0.004 | 0.680 |
| Weighted mode | 0.999 | 0.991 | 1.007 | 0.004 | 0.754 |
| MR Egger | 0.994 | 0.978 | 1.010 | 0.008 | 0.465 |
| Robust adjusted profile score (RAPS) | 0.999 | 0.995 | 1.004 | 0.003 | 0.833 |

**References**

1. Liu et al. *Nature Genetics* vol. 51 237–244 (2019).

2. Wootton et al. *Psychol. Med.* 1–9 (2019).
