# Supplementary material for: Little evidence for an effect of smoking on multiple sclerosis risk: A Mendelian Randomization study
Source: PLoS Biol. 2020 Nov 30;18(11):e3000973. doi: 10.1371/journal.pbio.3000973 (PMC7728259; doi:10.1371/journal.pbio.3000973)
Supplement: Attachment — Submitted filename: Response to reviewers .pdf [file pbio.3000973.s002.pdf]

**Response to peer-reviewers**

*We are grateful to the reviewers for their careful and constructive comments, which were helpful in improving our manuscript. Below is our point-by-point response to each of the comments.*

**Reviewer #1:** A sounds study that is suitable for publication in its current form if the editors believe it to carry sufficient priority.

*Response: We thank the reviewer for the kind comment.*

**Reviewer #2:** This is a revision of a manuscript describing the results of a study using mendelian randomization to study the effects of smoking on MS.
This is a well written paper and the results appear robust. However, I am not an expert in Mendelian randomization so I cannot speak to the veracity of the analytical methods employed.

I only have one suggested revision:

Line 205: why were you unable to stratify by smoking status? Please provide further explanation

*Response: We thank the reviewer for the kind comment. Ideally, we would have stratified on smoking status. However, in order to do this in the two-sample MR context would require a MS GWAS that is stratified by smoking status. In fact, smoking status was not available for participants in the MS GWAS. We have clarified this in the manuscript: 'in a two-sample MR context given that the MS GWAS is not stratified by smoking status', line 260-261.*

**Reviewer #3:**
Numerous observational studies and meta-analyses have suggested a causal relationship between smoking and MS risk, to the extent that it is considered a true risk factor amongst clinicians and MS researchers.

In this paper Mendelian randomization (MR) is used to assess the impact of smoking on the risk of MS. The critical assumptions as stated are: 1) the genetic variants robustly predict the exposure, 2) the genetic variants must not be associated with any confounders and 3) the genetic variants must only affect the outcome through the exposure.

While the results are negative, i.e. the authors find that there is no association between smoking and MS risk using MR, they are nonetheless important since this concept of smoking increasing risk of MS is firmly intrenched.

The paper is overall well-written, and the figures are displayed properly. The statistical methods are sound, and the conclusions follow from the results.

*Response: We thank the reviewer for their assessment of our work.*

It strikes me that the manuscript would benefit from slightly greater discussion of the Vandebergh study, ref 27, not simply as support but also comparing and contrasting methodology and sample populations.

*Response: Thank you for this suggestion. We have now expanded our discussion of the Vandebergh study and point out similarities and relevant differences: "That study similarly found that smoking initiation and lifetime smoking were not associated with increased MS risk using a two-sample MR approach within the same MS susceptibility GWAS. The authors also investigated the association between smoking heaviness and MS, although the interpretation of this exposure in the absence of stratification by smoking status is unclear. Indeed, stratification in this context is important as the effect of the smoking heaviness, proxied by the genetic instrument, should be examined among current smokers only, not never smokers. An important difference with our study is the additional sensitivity analyses performed herein to assess potential bias from pleiotropy, which is especially important given that pleiotropy can only be tested indirectly. Indeed, we obtained MR estimates excluding smoking-related variants in the MHC region due to its high potential for pleiotropy, in line with previous MR studies in MS*(1)*. We also report results from MR-RAPS and MR-PRESSO analyses. Consistent estimates across these additional pleiotropy robust MR methods increases the validity of our findings. Moreover, our analysis included an increased number of variants as we used an $r^2$ threshold of 0.8 instead of 0.9 for identifying proxies and did not exclude palindromic variants (given that all genetic datasets are on the same genome build), resulting in slightly narrower confidence interval." Lines 174-190*

The major criticism I have is that a positive control for the analysis would strengthen the conclusions. Please consider.

*Response: This is an excellent point and we agree with the review that positive controls using the genetic instruments that proxy our exposures would strengthen the conclusions. Previous studies from our group have shown that a polygenic risk score for genetic variants for smoking initiation predict smoking behaviour (self-report smoking initiation and ever e-cigarette use) in the Avon Longitudinal Study of Parents and Children*(2)*. Additionally, the lifetime smoking instrument, generated by our group, was validated in an independent sample and was associated with the positive controls of lung cancer, coronary heart disease and hypomethylation at the aryl-hydrocarbon receptor repressor site cg05575921*(3)*. This detail has been added to the methods section:*

*"A previous study from our group have shown that a polygenic risk score for genetic variants for smoking initiation predict smoking behaviour (self-report smoking initiation and ever e-cigarette use) in the Avon Longitudinal Study of Parents and Children*(2)*." Lines 409-412.*

*"This instrument, generated by our group, was validated in an independent sample and was associated with the positive controls of lung cancer, coronary heart disease and*

*hypomethylation at the aryl-hydrocarbon receptor repressor site cg05575921*(3).*" Lines 441-444.*

May I ask the authors to please expand on the validity or lack of validity of an odds ratio of 1.34 (figure 4). With the confidence interval shown the p value of .56 doesn't immediately make sense.
Also based on: Burgess S, Thompson SG. Interpreting findings from Mendelian randomization using the MR-Egger method Eur J Epidemiol. 2017;32(5):377-389. doi:10.1007/s10654-017-0255-x, may I ask the authors to comment on the idea:
"While the MR-Egger method is a worthwhile sensitivity analysis for detecting violations of the instrumental variable assumptions, there are several reasons why causal estimates from the MR-Egger method may be biased and have inflated Type 1 error rates in practice, including violations of the InSIDE assumption and the influence of outlying variants. The issues raised in this paper have potentially serious consequences for causal inferences from the MR-Egger approach."

*Response: We thank the reviewer for raising this concern. While the point estimate from the MR-Egger analysis of the effect of lifetime smoking on MS may appear to contradict the null finding from other analyses, we believe that this OR of 1.34 is simply due to the reduced power of MR-Egger and hence the very wide confidence intervals which still overlap the null (0.49-3.65). This method's lack of power is well documented, and because of this, the intercept estimate is more informative. In our analysis, this intercept was not different from zero (-0.003, p=0.69), which reduces the potential bias from directional pleiotropy. We have reformulated this sentence in the revised manuscript:*

*"While the MR-Egger estimate deviated somewhat from that of the IVW and other sensitivity analysis, this was explained by its reduced power as evidenced by the wide confidence intervals which still overlapped the null (OR 1.34, 95% CI 0.49 to 3.65)." Lines 143-146.*

*We also agree that the MR-Egger method has limitations and would be insufficient to exclude pleiotropy on its own. In fact, this underlies the rationale for presenting results from multiple MR pleiotropy robust methods in our study. This thorough assessment of pleiotropy is also the main difference between our and the Vandebergh study. To ensure that the readers are aware of the limitations of the MR-Egger method, we have now included a statement explain the InSIDE assumption in the methods:*

*"For instance, the MR-Egger method provides valid estimates even in the presence of pleiotropic effects as long as the size of these effects is independent of the effect of the genetic variants on the exposure (known as the InSIDE assumption)." Lines 320-323.*

For this section of the discussion: "Reverse causation could also partly explain the discrepancy between our MR results and 176 observational studies especially as MS onset may occur long before the first clinical 177 symptoms (39). For instance, this prodromal phase is characterized in part by a higher risk of 178 depression and anxiety up to 10 years prior to MS diagnosis (40), and these in turn are 179 associated with a higher rate of

smoking. This study sought to reduce bias from confounding 180 and reverse causation by using a MR design given genetic variants are much less associated 181 with confounders than directly measured environmental exposures (41) (here smoking) and 182 genetic variants are fixed over our lifetime ensuring directionality of effect. This is a major 183 strength of this study in establishing causality in the relationship between smoking and MS 184 risk.", can you please be more explicit about how/why observational studies and meta-analyses in particular are prone to reverse causation.

*Response: We have added in additional information clarifying reverse causation: "Reverse causality arises if preclinical aspects of a disease affect the risk factor, in this case, preclinical aspects of MS and their downstream effects (eg. stress) might influence the likelihood of a person smoking."* Lines 216-217.

Minor:
In addition, cigarette chemicals contribute mechanistically to MS……
In addition, cigarette chemicals may contribute mechanistically to MS……

*Response: This edit has been made.*

Recommend not abbreviating Instrumental variable assumptions to IV.

*Response: We have changed the wording to avoid the abbreviation.*

Poorly worded: "Furthermore, MR-PRESSO removes individual SNPs that contribute to heterogeneity disproportionately more than expected in order to reduce heterogeneity."

*Response: This has been reworded: 'Furthermore, MR-PRESSO removes individual outlier SNPs that contribute to heterogeneity disproportionately in order to correct for horizontal pleiotropy.' Lines 111-112.*

Reword: "These genetic variants facilitated epitope cross-reactivity and activation of T cells and smoking may strongly influence the risk of MS observed with these HLA genotypes." These genetic variants are thought to influence MS risk by facilitating epitope cross reactivity but this is an assumption.

*Response: This has been reworded: "Smoking status may strongly influence the risk of developing MS associated with these genetic variants. This is thought to be through facilitation of epitope cross-reactivity and subsequent activation of T cells." Lines 202-205.*

**Reviewer #4:** Observational studies have suggested that smoking is a risk factor for multiple sclerosis (MS). This paper used a two-sample Mendelian randomization approach to evaluate the causal relationship between smoking and MS risk. They used the most recent IMSGC summary statistics for MS risk and summary statistics from GWAS for lifetime smoking and smoking initiation. They do not find evidence of an effect of smoking on the risk of MS and conclude that it is unlikely that smoking is a contributor to MS. While I think

this study was generally well done, I do have some questions about the methodology and rigor of the analysis as well as some concerns related to the conclusions of the study.

*Response: We thank the reviewer for taking the time to assess our study.*

Did the other smoking phenotypes in the most recent smoking GWAS demonstrate similar effects (e.g. cigarettes per day) or conduct analyses assessing the association between age at smoking initiation and MS risk or age at MS onset or other smoking phenotypes like cotinine? Some MS risk factors may be particularly detrimental at certain age groups. Also, I realize that if only one of these additional phenotypes showed an effect, it may be a chance finding, but I think it would help the support the study's conclusions if a more comprehensive set of smoking phenotypes was considered. The study would also benefit if some justification of the specific phenotypes selected was provided.

*This is a valid consideration given the scope of our study. However, mendelian randomization analyses of smoking phenotypes such as heaviness (cigarettes per day), pack years, age at smoking initiation require stratifying the MS susceptibility GWAS on smoking status. Indeed, stratification in this context is important as the effect of these phenotypes, proxied by the genetic instrument, should be examined among current smokers only, not never smokers. Equally the effect of smoking on age at MS onset would indeed be interesting, however the MS GWAS did not include age at MS onset and therefore this analysis is currently not possible in a two-sample MR setting. We plan to perform these analyses if the appropriate GWAS become available.*

*We have clarified the specific phenotypes selected in the manuscript: "In order to examine the association between smoking and MS, we chose to investigate smoking behaviour using two specific phenotypes relating to the initial stage and a lifetime measure of tobacco use. Smoking initiation indicates whether an individual had ever smoked regularly and the lifetime smoking exposure which captures both smoking status (i.e. ever and never smokers), and among ever smokers takes into account smoking duration, heaviness and cessation. Due to lifetime smoking instrument including a score for non-smokers*(3)*, it enables the capture of smoking heaviness without needing to stratify on smoking status in the outcome GWAS as would be needed for other measures of smoking heaviness in a two-sample MR context. " Line 77-83.*

Are there overlapping participants in both the MS and smoking GWAS's (at least some of the controls from the MS study in the most recent GWAS? This should be at least discussed.

*Response: There is no sample overlap between the participants in the exposure and the outcome GWAS. We have clarified this in the discussion: "In as much as we could, we ensured that there was no sample overlap between participants (case and controls from the MS GWAS) in the exposure and outcome GWAS by using consortiums that comprised completely separate cohorts. Indeed, the MS GWAS did not include UK Biobank ensuring no overlap between lifetime smoking and MS GWAS cohorts. There is potential for overlap between the controls included in the smoking initiation GWAS and those of the MS GWAS, however this is likely to be small and therefore bias will be negligible*(4)*." Lines 212-217.*

Did the authors consider any interaction analyses with BMI, given that some smoking phenotypes are associated with BMI, though I realize this relationship is complex (as it could be for smoking/MS, but this is not considered or evaluated).

*Response: This is a complex relationship given that smoking has been shown to reduce BMI and having a higher BMI also makes you more likely to start smoking. Also, having a higher BMI leads to a greater risk of MS incidence. Given this complexity, including BMI in a multivariable MR would be interesting but beyond the scope of this current project. We had added to the discussion about this: "Smoking phenotypes are correlated with BMI and BMI has a causal effect on MS susceptibility*(5)*. This is an interesting and complex relationship. Multivariable MR allows for the adjustment of two correlated exposures, here smoking and BMI, investigating their effect on MS susceptibility and would be an interesting follow-up study. Vandebergh et al used this framework to show that BMI but not smoking initiation had a causal effect on MS risk*(6)*." Lines 239-243.*

While I agree that this analysis does not support smoking as, the MR confidence intervals are still quite large it's possible that the studies of MS are not large enough to detect such an effect.

*Response: While small effects are possible, a clinically relevant effect is unlikely given the tight confidence intervals, in particular for smoking initiation (0.92-1.16). This has been added to the discussion (line 192) and compared to the observational literature.*

Is the confidence interval for smoking cessation in the abstract correct? 1.03 (0.92, 1.61) - either the lower or upper bound seem incorrect.

*Response: Thank you for pointing out this typo. The confidence intervals should instead read (0.92, 1.16). We have corrected this in the abstract of the revised manuscript.*

This is minor, but I think it would be helpful to soften some of the claims in the introduction. For example, it's not definitive that there is a genetic interaction between HLA risk alleles, smoking and MS. As the authors mention in the Discussion, this was not replicated in other populations, so perhaps this should be revised here.

*Response: We thank the review for pointing this out. This has now been rephrased.*

*References:*


1. Harroud A, Morris JA, Forgetta V, Mitchell R, Smith GD, Sawcer SJ, et al. Effect of age at puberty on risk of multiple sclerosis: A mendelian randomization study. Neurology [Internet]. 2019 Apr 16 [cited 2020 Sep 14];92(16):E1803–10. Available from: https://pubmed.ncbi.nlm.nih.gov/30894442/
2. Khouja JN, Wootton RE, Taylor AE, Smith GD, Munafò MR. Association of genetic liability to smoking initiation with e-cigarette use in young adults. medRxiv [Internet]. 2020 Jun 12 [cited 2020 Sep 14];2020.06.10.20127464. Available from: https://www.medrxiv.org/content/10.1101/2020.06.10.20127464v1

3. Wootton RE, Richmond RC, Stuijfzand BG, Lawn RB, Sallis HM, Taylor GMJ, et al. Evidence for causal effects of lifetime smoking on risk for depression and schizophrenia: a Mendelian randomisation study. Psychol Med [Internet]. 2019 Nov 6 [cited 2020 Jan 20];1–9. Available from: http://www.ncbi.nlm.nih.gov/pubmed/31689377
4. Burgess S, Davies NM, Thompson SG. Bias due to participant overlap in two-sample Mendelian randomization. Genet Epidemiol [Internet]. 2016 Nov 1 [cited 2020 Sep 15];40(7):597–608. Available from: https://onlinelibrary.wiley.com/doi/full/10.1002/gepi.21998
5. Mokry LE, Ross S, Timpson NJ, Sawcer S, Davey Smith G, Richards JB. Obesity and Multiple Sclerosis: A Mendelian Randomization Study. Muraro PA, editor. PLOS Med [Internet]. 2016 Jun 28 [cited 2018 Sep 20];13(6):e1002053. Available from: http://dx.plos.org/10.1371/journal.pmed.1002053
6. Vandebergh M, Goris A. Smoking and multiple sclerosis risk: a Mendelian randomization study. J Neurol [Internet]. 2020 [cited 2020 Jun 22]; Available from: https://pubmed.ncbi.nlm.nih.gov/32529581/
